# Thrombotic Microangiopathic Hemolytic Anemia without Evidence of Hemolytic Uremic Syndrome

**DOI:** 10.4274/tjh.2015.0301

**Published:** 2016-02-17

**Authors:** Şinasi Özsoylu

**Affiliations:** 1 Retired Professor of Pediatrics, Hematology and Hepatology, Honorary Fellow of American Academy of Pediatrics, Honorary Member of American Pediatric Society

**Keywords:** Microangiopathy, Kidney functions, hemolytic anemia

## TO THE EDITOR

In a recent issue of this journal Dr. Oymak and her colleagues presented a clinically and genetically well-studied 5-year-old boy who was seen with severe microangiopathic hemolytic anemia without laboratory findings of renal involvement despite complement factor H gene mutations [1].

Because of Yeneral’s extensive review [2] on atypical hemolytic uremic syndrome (aHUS) published recently in the Turkish Journal of Hematology, I brought it to readers’ attention that more recently some authors do not use ‘aHUS’, which was historically used to distinguish heterogeneous uncharacterized syndromes from Shiga toxin-related HUS, since the term lacks both specificity and suggested causes [3].

Though in our patient with thrombotic thrombocytopenic purpura renal involvement was documented at the beginning but not in the last two recurrences, neither serum nor urinary findings indicated kidney involvement [4].

Although the discussions of Dr. Oymak et al. are well taken, the term ‘microangiopathic hemolytic anemia’ is covering the syndrome to a large extent as suggested by George and Nester [5].
